# A brief review of online education resources on gamification in addressing antimicrobial resistance

**DOI:** 10.1093/jacamr/dlad094

**Published:** 2023-08-08

**Authors:** Daniel Waruingi, Hafeez Hamza, Jonathan Babuya

**Affiliations:** School of Pharmacy, Jomo Kenyatta University of Agriculture and Technology, Nairobi, Kenya; School of Pharmacy, Girne American University, Girne, Cyprus; Faculty of Health Sciences, Busitema University, Tororo, Uganda

## Abstract

**Background:**

The burden of antimicrobial resistance (AMR) is projected to be highest in Africa, with a mortality of 99 per 100 000 deaths in sub-Saharan Africa. As a result, there is an urgent need to develop education resources to raise awareness and improve understanding of AMR.

**Aims:**

The aim of this review was to evaluate selected games, and inform regarding their suitability, and that of gamification in general in promoting education, and inspiring action against AMR.

**Objective:**

This brief review of online education resources seeks to inform on the use of games in promoting education on AMR by exploring different aspects of gamification such as accessibility, usability and playability with the delivery of desired learning outcomes. The insights obtained from the game helped inform recommendations and conclusions on how to best utilize gamification to deliver AMR education to target audiences.

**Methods:**

The games to be reviewed were selected using search terms ‘AMR Game’, ‘Antimicrobial Resistance Game’, ‘Antibiotic Resistance Game’, ‘ABR Game’, ‘Drug Resistance Game’ and ‘Superbugs Game’ in Google Play Store and Apple App Store search engines, given their positioning as suitable application software that house game applications. After applying a selection criterion, the number of selected games was narrowed to two: Micro-Combat and Terebra.

**Results:**

The games were analysed through four key factors: accessibility, usability, playability and learning outcomes. Both games were found to apply visual, written and aural game mechanics but Terebra had an increased touch with reality, enhancing its playability, due to the emphasis on aural triggers positioning AMR as a dire health threat. On the other hand, Micro-Combat was found to be more educative, and learning-oriented but less associated with reality, which negatively influenced its playability.

**Conclusions:**

In overall, gamification was found to be a good online resource to promote education on AMR through the review conducted on the two games, Terebra and Micro-Combat. Coupling gamification and conventional education mechanisms can go a long way in promoting the awareness and knowledge level of AMR among diverse populations The two games, Terebra and Micro-Combat, are great pilot AMR gamification projects that have set a good pace for utilization of games in AMR education. There is a need to develop AMR educational games that portray the reality in low- and middle-income countries, which was a bit lacking in the two games.

## Introduction

Antimicrobial resistance (AMR) is currently among the leading causes of death and morbidity in the world, with an estimated 1.27 million deaths in 2019 attributed to bacterial infections. In this study, the burden of AMR was projected to be highest in sub-Saharan Africa, with a mortality of 99 per 100 000 deaths.^1^ It is therefore prudent to develop educational resources that can be used to improve awareness and understanding of AMR in sub-Saharan Africa, and other low- and middle-income countries (LMICs). One of the objectives of the Global Action Plan on AMR adopted by the World Health Assembly is to improve awareness and understanding of AMR. This is through effective communication, education and training. Recently, there has been an increase in leveraging learning through the use of games, which involves conversion of learning concepts to suitable game designs contextualized to the target audience. It is commonly referred to as gamification.

Game-based learning has been found to improve performance and motivation in learning.^2^ A study conducted in Saudi Arabia reported that gamification had the potential to improve AMR knowledge with better retention than the conventional lectures.^3^ Games are a good educational avenue for different populations and can be harnessed not only to create awareness but also inspire action in the fight against AMR. However, for them to deliver on these two mandates, they have to be suitably designed. The aim of this review is to evaluate selected games, and gamification in general, to inform on its suitability in promoting education and inspiring action against AMR.

The objectives of this educational resource review are: (i) to investigate the different aspects of gamification such as accessibility, usability, playability, and learning outcomes in the games selected, and their role in promoting learning; and (ii) to recommend, and draw possible conclusions about, gamification as an avenue for creating awareness and inspiring action in the fight against AMR.

## Methods

The games to be reviewed were selected using search terms ‘AMR Game’, ‘Antimicrobial Resistance Game’, ‘Antibiotic Resistance Game’, ‘ABR Game’, ‘Drug Resistance Game’ and ‘Superbugs Game’ in Google Play Store and Apple App Store search engines, given their positioning as commonly used software that house game applications. A total of six games were identified in the first stage (three games from Google Play Store: Terebra, Private Penny and Micro-combat; and three games from Apple App Store: Bacteria Merger, Phage Invaders and Dermis Defense).

**Figure 1.**
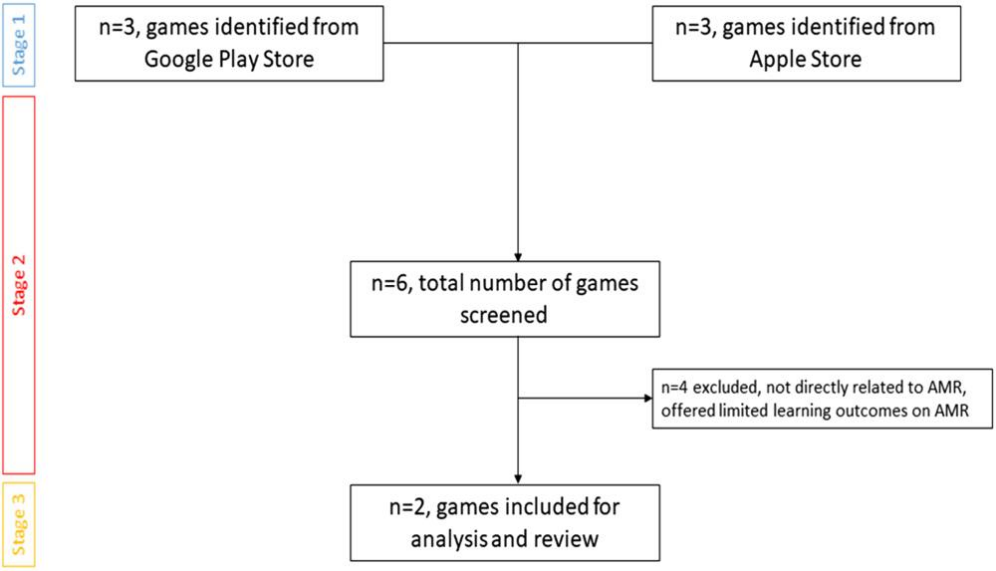
Flow diagram for game selection.

**Figure 2.**
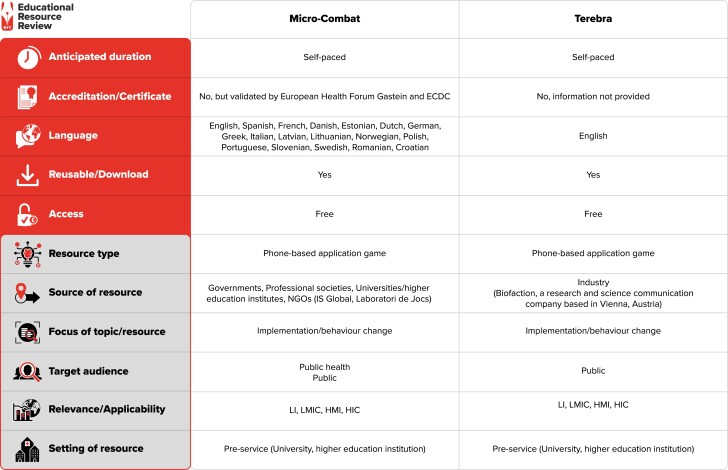
Summary of the resources reviewed. Resource web link(s): Micro-Combat: https://microcombat.eu/#/, https://apps.apple.com/gb/app/micro-combat/id1516625163; Terebra: https://play.google.com/store/apps/details?id=com.biofaction.synpeptide&hl=en_US&gl=US, https://apps.apple.com/us/app/terebra/id1259787366. WHO region and country of developer (World Bank classification): Micro-Combat: European Region, Spain (HIC); Terebra: European Region, Austria (HIC). The full classification scheme is available at http://bsac.org.uk/wp-content/uploads/2019/03/Educational-resource-review-classification-scheme.pdf. LI, low-income countries; LMIC, low- and middle-income countries; HMI, high- and middle-income countries; HIC, high-income countries.

In the second stage, the screening of the six games was conducted, and four of the games were eliminated from consideration since they were not directly connected to AMR and provided very limited learning outcomes on the subject. The third and final stage involved an analysis of the games selected. This has been illustrated as a flow diagram in Figure 1. A summary of Micro-Combat and Terebra has been offered in Figure 2.

## Results and discussion

The effectiveness of learning from a game can be seen as a collective measure of accessibility, usability, playability and learning outcomes.^4^ These three key factors, alongside the accessibility of the game, were applied in reviewing the games selected. The two games selected were Terebra and Micro-Combat. Terebra is an individual puzzle game where the player assumes the role of a scientist racing against severe drug-resistant epidemics by creating new antibiotics. The faster the player develops a new antibiotic the larger the population of the world saved. The game becomes more challenging as you go up the levels as the bacteria become more resistant. On the other hand, Micro-Combat is a card cooperative game where the players assume the role of healthcare personnel preventing infection by pathogenic agents through the use of preventive measures and medication.

### Accessibility

Both games are freely accessible via Google Play Store or Apple App Store, and their development was funded. Micro-Combat has a higher number of downloads (1000+ category) compared with Terebra’s 100+ category. This is attributable to the intentional marketing strategy employed in Micro-Combat. No information was found regarding a marketing strategy for Terebra. Terebra is compatible with a lower version of the operating system and was released earlier in 2017 compared with Micro-Combat’s release in 2020. Micro-Combat is available in 19 languages while Terebra is only available in English, making it inaccessible for non-English-speaking audiences. Terebra is classified as a puzzle game rated for 12+ years while Micro-Combat is classified as a card game rated 3+ in Play Store but rated as 10+ years on the game’s website. Both games are therefore suitable for diverse populations.

### Usability

The usability of a game involves the level of ease with which the player understands and plays, promoting their immersion in the game.^2^ Micro-Combat has a personalized account system enabling cooperation between different players. This can help track attendance, harness problem-solving skills, and improve ownership of AMR challenges.^5^ On the other hand, Terebra is a puzzle game with simple instructions. Puzzles have been found to foster problem-solving, analytical and memory skills. Game-based learning has also been found to be influenced by instructional strategies.^6^ Here, we looked at the location and nature of instructions. Micro-Combat’s instructions are inbuilt into the game itself while Terebra’s instructions are sequestered in the Google Play game description rather than inside the game, making it difficult for a player to locate them, which could be disappointing and negatively affect engagement in the game. Both games have audio-visual and written instructions. From experience in playing the game, the authors found that it was easy to miss out on Terebra’s instructions, which could affect the player’s motivation. Micro-Combat’s instructions were also difficult to comprehend compared with Terebra’s. Difficult-to-understand instructions can undermine a player’s understanding of the game. Overall, Terebra had better usability compared with Micro-Combat. The usability of a game helps the player understand how to navigate the game, and therefore is an important aspect to review when evaluating utilization of games as education resources. If a player finds it difficult to understand how to play a game, it is likely that they will be disappointed, and spend less time engaging in the game, which negatively affects the intended learning outcomes.

### Playability

The playability of a game involves a game’s educational capability while still retaining its immersive capacity and motivation.^7^ There are different frameworks used to evaluate the playability of the game but the background lies in the challenge, mastery and reward mechanisms of the game.^8^ This guides the design of the game and influences the level of immersion the player experiences.

Micro-Combat's game challenge lies in the use of limited preventive measures and medications to prevent characters from getting infections, while Terebra’s challenge is developing a new peptide antibiotic in a drug-resistant epidemic setting amidst a fast-declining world population. Both games address the current threat of AMR but from different perspectives; Micro-Combat addresses the need to improve disease prevention measures in reducing spread of infections, while Terebra focuses on development of novel antimicrobials to avert the drug resistance crisis. Both games apply visual, written and aural game mechanics but Terebra has an increased touch with reality due to the emphasis on aural triggers such as sirens, helicopter evacuations, wails and emergency news briefs, which insinuate a serious pandemic, and trigger the need for urgent action. However, it is less educative on ways to act to avert AMR. On the other hand, Micro-Combat is educative with a broad perspective on processes leading to the development and spread of drug-resistant infections and ways to act, such as potential mitigation strategies that can be employed. The limited time for intervention in each game promotes a higher demand for auditory, visual and mental attention, promoting immersion in the game.

Games can have both extrinsic and intrinsic motivation, which is important in maintaining optimal concentration, and engagement, which is crucial in promoting long-duration playing of the game. Ultimately, more time spent in playing the game is likely to improve the learning outcomes from the game.^9^ Extrinsic motivation stems from behaviour that is pegged on the desire to attain an outcome that is separate from the action itself. On the other hand, intrinsic motivation refers to engaging in satisfying behaviour that is independent of external incentives. Both intrinsic and extrinsic motivation have a huge influence on the gaming experience. In both Terebra and Micro-Combat, intrinsic motivation arises from the feeling of responsibility to alleviate the challenges expressed in the game setup. In both games, one feels the nudge to act to prevent the looming AMR crisis projected in the game’s design. In Terebra one feels intrinsically motivated to develop new antibiotics, while in Micro-Combat there is a nudge for continued playing to ensure better infection prevention and therapeutic interventions to avert AMR.^9^ Micro-Combat exhibits extrinsic motivation through score rewards and trophies, but Terebra has no incentives.

### Learning outcomes of the games

Learning outcomes of a game evaluate the game’s capability to promote education, in this case education on AMR. Micro-Combat promotes learning of scientific concepts on development, the spread of drug-resistant infection, and alleviation measures that can be employed. However, there is less focus on issues such as systemic challenges, and behavioural tendencies that promote irrational use of antimicrobials. The less specific focus in Micro-Combat may undermine positioning of major enablers of AMR as intended game’s learning outcome, especially for audiences from LMICs.

Terebra stirs emotions and positions AMR as a dire health threat. This is likely to have both positive and negative outcomes. It can facilitate the retention of information for a longer period but it could also undermine the credibility of information shared, and discourage the need to take action due to its apocalyptic nature. Terebra does not address the drivers of AMR, and the development of new antibiotics is postulated as the magic bullet to solving AMR, which is not the case in reality. Poor access to already existing antimicrobials, irrational use of the antimicrobials, and poor infection prevention and control strategies are among major drivers of AMR, especially in LMICs.^10^ These AMR drivers thrive due to poor health systems and the poor purchasing power of many LMICs. The AMR drivers are not addressed in the game, and this could undermine players’ inspiration to initiate action on mitigating AMR after playing the game, especially if there are from LMICs.

The authors spent an average of 15 min playing one game of Terebra before losing and restarting again. They had an average of four replays for two sessions of playing the game. At the beginning, the duration of play was short before the players understood the game well but the duration increased with time as they got to understand the game better. On the other hand, it took them an average of 25 min to play Micro-Combat, with an average of four replays in two sessions. Despite their different game designs, both games were generally challenging but exciting.

## Recommendations and conclusions

Overall, gamification was found to be a good online resource to promote education on AMR through the evaluation conducted on the two games, Terebra and Micro-Combat. Generally, the two games are great pilot AMR gamification projects, which have set a good pace for utilization of games in AMR education. However, for both games, it is important to consider a good balance in the diversity of the information shared to ensure there is an envisioned targeted learning outcome from the games. Educative games should also have the capability of inspiring action or positive change among the players. Intentional efforts should be made to ensure reach of the games to a wider target population, and adaptation to diverse contexts. An evaluation strategy is also important to inform on the attainment of the learning outcomes and recommend potential future improvements in utilization of gamification in AMR education. We recommend further in-depth reviews of these two games reviewed in this paper, and other existing AMR games, to determine the potential and significance of gamification and game-based learning in AMR education and inspiring action. The rapid mobile connectivity and internet penetration in sub-Saharan Africa, and other LMICs, positions gamification as a strategic tool in promoting education of AMR in these settings. There is a need to develop educational games that portray the reality of AMR in LMICs. Both Micro-Combat and Terebra have been developed in high-income countries, which makes the relationship with the situation in LMICs quite distant. Lastly, we recommend initiation of studies that evaluate coupling of gamification and conventional education mechanisms in promoting the awareness and knowledge level of AMR among different populations.
